# *In vivo* analgesic, antipyretic, and anti-inflammatory potential in Swiss albino mice and *in vitro* thrombolytic activity of hydroalcoholic extract from *Litsea glutinosa* leaves

**DOI:** 10.1186/0717-6287-47-56

**Published:** 2014-10-29

**Authors:** Rumpa Bhowmick, Md Shahid Sarwar, Syed Masudur Rahman Dewan, Abhijit Das, Binayok Das, Mir Muhammad Nasir Uddin, Md Siddiqul Islam, Mohammad Safiqul Islam

**Affiliations:** Department of Pharmacy, Noakhali Science and Technology University, Sonapur, Noakhali, 3814 Bangladesh; Department of Pharmacy, Southeast University, Dhaka, 1213 Bangladesh; Department of Oncology, Ashic Oncology and Palliative Care Center, Dhaka, 1207 Bangladesh; Department of Pharmacy, University of Chittagong, Chittagong, 4331 Bangladesh; Department of Pharmacy, Manarat International University, Dhaka, 1216 Bangladesh

**Keywords:** Acetic acid induced writhing, Analgesic, Anti-inflammatory, Antipyretic, *Litsea glutinosa*, Thrombolytic

## Abstract

**Background:**

The study was conducted to evaluate the *in vitro* thrombolytic activity, and *in vivo* analgesic, anti-inflammatory and antipyretic potentials of different hydrocarbon soluble extracts of *Litsea glutinosa* leaves for the first time widely used in the folkloric treatments in Bangladesh. This work aimed to create new insights on the fundamental mechanisms of the plant extracts involved in these activities.

**Results:**

In thrombolytic activity assay, a significant clot disruption was observed at dose of 1 mg/mL for each of the extracts (volume 100 μL) when compared to the standard drug streptokinase. The n-hexane, ethyl acetate, chloroform, and crude methanolic extracts showed 32.23 ± 0.26, 37.67 ± 1.31, 43.13 ± 0.85, and 46.78 ± 0.9% clot lysis, respectively, whereas the positive control streptokinase showed 93.35 ± 0.35% disruption at the dose of 30,000 I.U. In hot plate method, the highest pain inhibitory activity was found at a dose of 500 mg/kg of crude extract (15.54 ± 0.37 sec) which differed significantly (P <0.01 and P <0.001) with that of the standard drug ketorolac (16.38 ± 0.27 sec). In acetic acid induced writhing test, the crude methanolic extract showed significant (P <0.01 and P <0.001) analgesic potential at doses 250 and 500 mg/kg body weight (45.98 and 56.32% inhibition, respectively), where ketorolac showed 64.36% inhibition. In anti-inflammatory activity test, the crude methanolic extract showed significant (P <0.001) potential at doses 250 and 500 mg/kg body weight (1.51 ± 0.04 and 1.47 ± 0.03 mm paw edema, respectively), where ketorolac showed 1.64 ± 0.05 mm edema after 3 h of carrageenan injection. In antipyretic activity assay, the crude extract showed notable reduction in body temperature (32.78 ± 0.46°C) at dose of 500 mg/kg-body weight, when the standard (at dose 150 mg/kg-body weight) exerted 33.32 ± 0.67°C temperature after 3 h of administration.

**Conclusions:**

Our results yield that the crude hydroalcoholic extract has better effects than the other in all trials. In the context, it can be said that the leaves of *L. glutinosa* possess remarkable pharmacological effects, and justify its traditional use as analgesic, antipyretic, anti-inflammatory, and thrombolytic agent.

## Background

Traditional knowledge regarding medicinal plants and their use by indigenous cultures is not only useful for maintenance of cultural traditions and biodiversity but also for community healthcare and drug development in the present and future. Therapies with synthetic tropical applications have many side effects and cannot be afforded by the people due to higher cost of the drug. For overcoming this problem plants growing around us are utilized without scientific validation. The use of higher plants and their extracts to treat infections is an age-old practice. Traditional medicinal practice has been known for centuries in many parts of the world. Herbal medicines are gaining interest because of their cost effective and eco-friendly attributes [1].

*L. glutinosa* belongs to the family Lauraceae and is a well-known evergreen species growing wild in the forest of Chittagong and Sylhet districts in Bangladesh. It is occasionally planted in most areas of the country [2]. Leaves are mucilaginous and considered for antispasmodic, emollient, and poultice. The leaves are also used in diarrhea and dysentery as well as for the treatment of wounds and bruises [2]. The leaves were reported for the treatment of the spontaneous and excessive flow of semen in young boys [3]. The leaf extract also shows antibacterial and cardiovascular activities [4]. The berries yield oil which is used by some tribal practitioners in the treatment of rheumatism. Tannin, β-sitosterol, and actinodaphnine are reported to be the common constituents of the species; and other constituents known are: Boldine, norboldine, laurotetanine, n-methyllaurotetanine, n-methylactinodaphnine, quercetin, sebiferine, litseferine etc. [5].

Thrombosis is the fundamental pathophysiological process that underlies the acute coronary disorders such as pulmonary emboli, deep vein thrombosis, strokes and heart attacks; which are the main causes of morbidity and mortality in developed countries [6]. This disease is characterized by the development of a blood clot (thrombus) in the circulatory system of the body due to the failure of homeostasis which leads to vascular blockade and while recovering causes fatal consequences, such as myocardial or cerebral infarction, as well as death [7]. Therefore, anticoagulation therapy is the basis of management, and the proper choice of thrombolytic drugs [8].

Pain is an unpleasant sensory and emotional experience associated with actual or potential tissue damage [9]. By acting in the CNS or on the peripheral pain mechanism, analgesic compounds selectively relieves pain without significant alteration of consciousness. Actually analgesics are applied when the noxious stimulus cannot be removed or as adjuvant to more etiological approach to pain [10].

Inflammation is the response of living tissues to injury. It involves a complex array of enzyme activation, mediator release, and extravasations of fluid, cell migration, tissue breakdown and repair. Non-steroidal anti-inflammatory drugs (NSAID) are among the most commonly prescribed drugs due to their consistent effectiveness in the treatment of pain, fever, inflammation and rheumatic disorders. However, their use is associated with adverse effects at the level of digestive tract, ranging from dyspeptic symptoms, gastrointestinal erosions and peptic ulcers to more serious complications, such as over bleeding or perforation [11]. Therefore to overcome the toxicity of NSAID, the development of new anti-inflammatory drugs is still necessary and the natural product such as medicinal plants could lead in discovering new anti-inflammatory drugs with less undesirable effects [12].

Pyrexia or fever is usually caused as a secondary impact of infection, tissue damage, inflammation, graft rejection and malignancy or other diseased states. The body by its natural defense mechanism creates an environment where infectious agent or damaged tissue cannot survive. Generally, infected or damaged tissue initiates the enhanced formation of pro-inflammatory mediators (cytokines like interleukin 1β, α, β and TNF-α) which increase the synthesis of prostaglandin E_2_ (PGE_2_) near preoptic hypothalamus area and thereby triggering the hypothalamus to elevate the body temperature [13]. Most of the antipyretic drugs normally prevent or inhibit COX-2 expression to reduce the elevated body temperature by inhibiting PGE_2_ biosynthesis. Moreover, these synthetic agents irreversibly inhibit COX-2 with high selectivity which is toxic to the hepatic cells, glomeruli, cortex of brain and heart muscles, whereas natural COX-2 inhibitors usually have lower selectivity with fewer side effects [14]. *L. glutinosa* was selected due to its availability in Bangladesh, therefore, used in rural areas for different treatments, and not such investigations have been carried out with this plant native to Bangladesh. Our main goal was to evaluate the in vivo antipyretic, anti-inflammatory and analgesic activity, and investigate the thrombolytic property of the plant leaves to validate its traditional uses.

## Results

### Thrombolytic activity test

**Figure 1:**
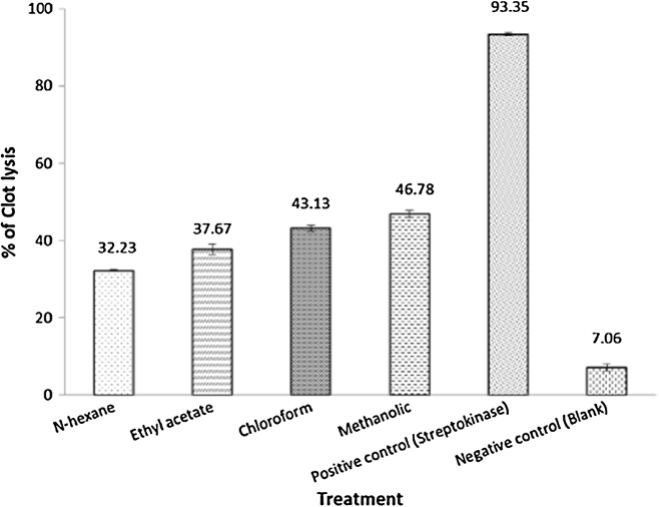
**Thrombolytic effect of hydroalcoholic extracts of** ***L. glutinosa*** **leaves.**

The effective clot lysis percentage by four different extracts of the plant, positive thrombolytic control (Streptokinase) and negative control (water) is tabulated in Figure 1. From the Figure 1, it is evident that the percentage of clot lysis was 93.35 ± 0.35% when 100 μl of streptokinase (30,000 I.U.) was used as a positive control, while in case of negative control (water) the percentage of clot lysis was negligible (7.06 ± 0.95%). Among different extracts (concentration 1 mg/ml), the crude methanolic extract showed highest activity (46.78 ± 0.90%), whereas n-hexane soluble fraction showed the lowest activity (32.23 ± 0.26%), which was much higher than the negative control (water).

### Analgesic activity test

#### Hot plate test

Two doses of methanolic extract of leaves of *L. glutinosa* increased the animal (Swiss-albino mice) reaction time to the thermal stimulus which has been summarized in Table 1. The highest pain inhibition of thermal stimulus was found at a higher dose 500 mg/kg of crude extract which exhibited maximum time for the response against thermal stimuli (15.54 ± 0.37 sec) that is comparable to ketorolac (16.38 ± 0.27 sec) and found statistically significant (P <0.001 and P <0.01) when compared to both the control and the standard drug.

**Table 1:** **Effect of hydroalcholic extracts of** ***L. glutinosa*** **leaves on hot plate test**

Treatment	Response time (sec)				
	0 h	0.5 h	1 h	2 h	3 h
Control (10 ml/kg)	8.30 ± 0.63	7.42 ± 0.42	7.50 ± 0.39	7.50 ± 0.45	7.18 ± 0.33
Standard (10 mg/kg)	8.42 ± 0.29	11.59 ± 0.32^**^	13.58 ± 0.38^**^	14.98 ± 0.48^***^	16.38 ± 0.27^***^
n-hexane extract (250 mg/kg)	8.27 ± 0.32	10.25 ± 0.31^**c^	10.94 ± 0.13^***a^	11.27 ± 0.58^**a^	11.98 ± 0.37^***b^
n-hexane extract (500 mg/kg)	8.57 ± 0.63	11.01 ± 0.66^**^	11.43 ± 0.25^***b^	11.59 ± 0.76^**b^	12.22 ± 0.48^***a^
Ethyl acetate extract (250 mg/kg)	8.73 ± 0.30	10.05 ± 0.32^**a^	10.30 ± 0.74^**a^	11.13 ± 0.47^**b^	11.39 ± 0.65^**b^
Ethyl acetate extract (500 mg/kg)	8.70 ± 0.56	10.87 ± 0.46	11.36 ± 0.27^***b^	11.48 ± 0.59^**b^	11.89 ± 0.63^**b^
chloroform extract (250 mg/kg)	8.90 ± 0.40	10.58 ± 0.29^***c^	11.03 ± 0.40^**b^	11.33 ± 0.66^*b^	12.07 ± 0.28^***a^
chloroform extract (500 mg/kg)	8.87 ± 0.15	11.16 ± 0.47^**^	11.49 ± 0.24^***b^	11.69 ± 0.78^**b^	12.31 ± 0.54^***b^
Methanolic extract (250 mg/kg)	9.10 ± 0.33	11.48 ± 0.63^**^	13.20 ± 0.38^**^	14.24 ± 0.42^***^	15.06 ± 0.36^***^
Methanolic extract (500 mg/kg)	8.82 ± 0.18	11.54 ± 0.52^**^	13.38 ± 0.44^**^	14.56 ± 0.49^***^	15.54 ± 0.37^***b^

Values are expressed as mean ± SEM (Standard error mean); Values are calculated using one-way ANOVA followed by Dennett’s test; ^***^indicates P <0.001 and ^**^indicates P <0.01 when compared to control; ^a^indicates P <0.001, ^b^indicates P <0.01 and ^c^indicates P <0.05 when compared to standard drug; p.o.; n = 6.

#### Acetic acid induced writhing test

The effect of the methanolic extract of *L. glutinosa* leaves on acetic acid induced writhing in mice is given in Table 2. At the dose of 500 mg/kg of body weight, the crude extract produced 56.32% writhing inhibition in test animals. The results were statistically significant (P <0.001) compared to the negative control.

**Table 2:** **Effect of hydroalcholic extracts of** ***L. glutinosa*** **leaves on acetic acid induced writhing in mice**

Treatment	Total writhing count (Mean ± SEM)	% Inhibition
Control	17.4 ± 2.50	**-**
Standard	6.2 ± 0.66***	64.36
n-hexane extract (250 mg/kg)	10.10 ± 0.41**	41.95
n-hexane extract (500 mg/kg)	8.7 ± 0.57***	50.00
Ethyl acetate extract (250 mg/kg)	10.14 ± 0.63**	41.72
Ethyl acetate extract (500 mg/kg)	9.96 ± 0.29**	42.76
Chloroform extract (250 mg/kg)	11.6 ± 0.31**	33.33
Chloroform extract (500 mg/kg)	9.15 ± 0.36**	47.41
Methanolic extract (250 mg/kg)	9.40 ± 0.51**	45.98
Methanolic extract (500 mg/kg)	7.60 ± 0.51***	56.32

Values are expressed as mean ± SEM (Standard error mean); ***indicates P <0.001, and **indicates P <0.01; one-way ANOVA followed by Dennett’s test as compared to control.

### Anti-inflammatory activity test

The anti-inflammatory effect of the crude methanolic extract using carrageenan induced oedema tests is expressed in Table 3. In this test, the positive control (ketorolac) significantly (P <0.05; P <0.001) decreased the paw edema volume, 0.96 ± 0.05 to 1.64 ± 0.05 mm, at 1 to 3 h after carrageenan injection compared to control (saline) with edema volume. A maximum edema paw volume of 1.78 ± 0.03 mm was observed in the control mice, after 3 h of the carrageenan injection. Mice administered with extract at 500 mg/kg body weight significantly decreased (P <0.05; P <0.01) the carrageenan-induced edema paw volume from 1 to 3 h compared to the control (saline) at a dose of 2 ml/kg body weight. The highest reduction in the paw volume by the 500 mg/kg body weight was 1.47 ± 0.03 mm when compared to that of the control (saline) (1.78 ± 0.03 mm) at 3 h.

**Table 3:** **Anti-inflammatory activity of hydroalcholic extracts of leaves of** ***L. glutinosa***

Treatments	Paw volume (mm)			
	0 h	1 h	2 h	3 h
Control	0.73 ± 0.04	1.31 ± 0.19	1.58 ± 0.02	1.78 ± 0.03
Standard	0.69 ± 0.04	0.96 ± 0.05^**^	1.34 ± 0.05^***^	1.64 ± 0.05^***^
n-hexane extract (250 mg/kg)	0.90 ± 0.14	1.91 ± 0.04^**^	2.02 ± 0.04^**^	2.19 ± 0.03^***^
n-hexane extract (500 mg/kg)	0.71 ± 0.05	1.60 ± 0.02^**^	1.98 ± 0.13^**^	2.17 ± 0.04^***^
Ethyl acetate extract (250 mg/kg)	0.91 ± 0.11	1.11 ± 0.05^**^	1.51 ± 0.03^***^	1.32 ± 0.03^**^
Ethyl acetate extract (500 mg/kg)	0.88 ± 0.02	1.41 ± 0.07^**^	1.63 ± 0.10^**^	1.77 ± 0.02^***^
Chloroform extract (250 mg/kg)	0.81 ± 0.04	1.21 ± 0.06^**^	1.42 ± 0.02^**^	1.59 ± 0.01^***^
Chloroform extract (500 mg/kg)	0.69 ± 0.08	1.00 ± 0.03^**^	1.40 ± 0.06^**^	1.47 ± 0.03^***^
Methanolic extract (250 mg/kg)	0.77 ± 0.04	1.01 ± 0.04^**^	1.42 ± 0.03^**^	1.51 ± 0.04^***^
Methanolic extract (500 mg/kg)	0.69 ± 0.05	1.00 ± 0.04^**^	1.40 ± 0.03^**^	1.47 ± 0.03^***^

Values are expressed as mean ± SEM (Standard error mean); Values are calculated as compared to control using one way-ANOVA followed by Dunnet’s Test; **indicates P <0.05; ***indicates P <0.001 vs. control; n = 5.

### Antipyretic activity test

Effect of different extracts of *L. glutinosa* on rectal temperature in mice is presented in Table 4. The subcutaneous injection of yeast suspension markedly elevated the rectal temperature after 18 h of administration. Treatment with the crude methanolic extract showed significant (P <0.05; P <0.001) activity against induced pyrexia when compared with the control treatment.

**Table 4:** **Effect of the hydroalcholic extracts of** ***L. glutinosa*** **on yeast-induced pyrexia in mice**

Treatment	Initial	Pyretic	1 h	2 h	3 h
Control (10 ml/kg)	33.33 ± 0.44	35.70 ± 0.46	35.66 ± 0.44	35.77 ± 0.56	35.39 ± 0.66
Standard (150 mg/kg)	33.28 ± 0.42	35.73 ± 0.55	34.80 ± 0.68	34.20 ± 0.63^**^	33.32 ± 0.67^***^
n-hexane extract (500 mg/kg)	33.80 ± 0.30	36.17 ± 0.40	35.90 ± 0.46	35.77 ± 0.25^c^	35.60 ± 0.17^b^
Ethyl acetate extract (500 mg/kg)	33.69 ± 0.79	35.57 ± 0.25	35.27 ± 0.15	35.00 ± 0.36^*^	34.87 ± 0.32^c^
Chloroform extract (500 mg/kg)	33.43 ± 1.37	35.37 ± 0.60	35.45 ± 0.70	35.23 ± 0.21^c^	35.17 ± 0.32^b^
Methanolic extract (500 mg/kg)	33.64 ± 0.80	36.93 ± 0.54	36.26 ± 0.68^*c^	34.13 ± 0.54^**^	32.78 ± 0.46^***^

Values are expressed as mean ± SEM (Standard error mean); Values are calculated using one-way ANOVA followed by Dennett’s test; ^***^indicates P <0.001, ^**^indicates P <0.01 and ^*^indicates P <0.05 when compared to control; ^b^indicates P <0.01and ^c^indicates P <0.05 when compared to standard drug; n = 5.

## Discussion

Thrombosis or blood clot formation is a critical event in which the damaged regions of the endothelial cell surface or blood vessel are blocked by the deposition of platelets, tissue factor and fibrin [15]. In the formation process the major role is played by platelets as the process of thrombosis is initiated when the activated platelets form platelets to platelets bonds. These activated platelets further bind to the leucocytes and bring them into a complex process of plaque formation and growth [16]. It is the thrombolytic agents that lyse clot by disrupting the fibrinogen and fibrin contained in a clot. Plasmin is one of the natural anti-thrombotic agents. The cell surface bound plasminogen is easily activated to plasmin which ultimately leads to fibrinolysis [17]. After a long process of trial and error the scientists have discovered several thrombolytic drugs from various sources. To make those drugs more site specific and effective some of them have been modified with modern recombinant technology. Streptokinase (a bacterial plasminogen activator), a widely used thrombolytic agent, is capable of converting additional plasminogen to plasmin. But this drug has several adverse effects like bleeding and embolism which lead to further complications. To overcome these complications a number of studies have been conducted by various researchers in order to discover new sources of herbs and natural foods and their supplements having antithrombotic effect with minimal adverse effect [6]. As a part of that research work we also tried to find whether the herbal preparations of *L. glutinosa* leaves possess clot lysis potentiality or not. When we compared the result of positive control (streptokinase) with that of negative control (water), we found that there was negligible amount of clot disruption when water was added to the clot. This prominent result encouraged us to compare four different test samples in the same manner against the negative control and observe significant thrombolytic activity. It was reported that phytochemicals like saponin, alkaloids and tannin are responsible for thrombolytic activity [18]. As the bark extract of *L. glutinosa* possesses saponin, alkaloids [19], therefore the possibility of the presence of these phytochemicals in the leaves extract may be the probable reason of demonstrating the thrombolytic activity.

It was observed from the study that in both analgesic activity assay models the plant extract demonstrated analgesic effects. This means that the extract may possess both peripheral and central analgesic effects. The leaves extract of *L. glutinosa* exhibited significant dose dependent inhibition of acetic acid‒induced writhing in mice in comparison to that of the control (saline). Acetic acid induces inflammatory pain by impelling capillary permeability [20], and releasing substances that excite pain nerve endings [21]. The peripheral analgesic effect is generally mediated by the NSAIDs through inhibition of cyclooxygenase and/or lipoxygenase (and other inflammatory mediators) or inhibition of pain responses mediated by noiceptors peripherally [22]. Therefore, it is possible that crude extract of *L. glutinosa* leaves may be showing analgesic effect through these mechanisms although the exact mechanism of action is yet to be discovered. Again in hot plate test the extract also showed prominent antinociceptive effect against the standard drug ketorolac. The hot plate response is a more complex supraspinally organized behavior [23]. The μ receptor is a type of receptor that is commonly known as the pain relief receptor. It has been proved to be a potent receptor in regulating thermal pain [24]. Moreover, activation of μ_2_ opioid subtype receptor leads to spinal analgesia [25]. Therefore, by considering the test report, it may be assumed that the antinociceptive activity of *L. glutinosa* leaves extract is likely to be mediated centrally although the exact mechanism is yet to be discovered. Previous studies on different plant extracts showed analgesic effect in animal models and their effects have been attributed to the presence of alkaloids, glycosides, flavonoids and saponins [26, 27]. The phytochemical screening of *L. glutinosa* extract revealed the presence of most of the above‒mentioned phytochemicals [20]. Thus it is possible that the analgesic activity of *L. glutinosa* leaves extract can be due to the presence of these phytochemical constituents.

The present study was also conducted to evaluate the probable anti-inflammatory activity of the methanol extract of *L. glutinosa* leaves on mice in an acute inflammatory model. The carrageenan‒induced mice paw oedema is a suitable test for evaluating anti‒inflammatory drugs or natural products [28]. Carrageenan induced paw edema is biphasic event. In the first phase there are incidences like release of histamine, serotonin and kinins whereas the second phase of edema is imposed by release of prostaglandins, protease and lysosome. The second phase is sensitive to most clinically effective anti-inflammatory drugs [29]. The results of present study showed that the role of methanolic extract of *L. glutinosa* leaves against carrageenan induced acute inflammation was significant. The leaves extract at a dose of 250 and 500 mg/kg showed significant dose dependent reduction in paw size from 1–4 h and elicited anti‒inflammatory response comparable with standard drug ketorolac. This might be due to the inhibition of the biphasic response induced by the carrageenan. Therefore, it is possible that acute anti‒inflammatory effect of *L. glutinosa* leaves may involve multiple mechanisms like inhibition of either cyclooxygenase and/or lypooxygenase enzyme or inhibition of synthesis, release and action of above inflammatory mediators; but the exact mechanism of action needs to be discovered by further investigation.

It is known that fever is provoked by many exogenous substances in animal models, including bacterial endotoxins and microbe infection. These exogenous pyrogens are responsible for the production of different pro-inflammatory cytokines which stimulate the release of local prostaglandins (PGs) by entering into the hypothalamic circulation and therefore resetting the hypothalamic thermal set point. Thus to regulate the body temperature it is necessary to have a delicate balance between the production and loss of heat. It is the hypothalamus that regulates the set point and also controls body temperature. The non-steroidal anti-inflammatory drugs inhibit the prostaglandin synthetase within the hypothalamus and thereby demonstrate their antipyretic action [30]. The present study revealed that the methanolic extract of *L. glutinosa* leaves causes a significant antipyretic effect in yeast provoked elevation of body temperature. Here, the methanolic extract caused almost similar result in lowering of body temperature, in comparison to that of standard drug. It may be predicted that the extract their antipyretic action through inhibition of prostaglandin synthetase within the hypothalamus as like as the NSAIDs. Although, there is no direct evidence of *L. glutinosa* to interfere with prostaglandin synthesis in hypothalamus but it can be supported by a related study in which *Dalbergia odorifera* extract was found to inhibit prostaglandin biosynthesis [31]. Thus the present pharmacological evidence provides support for the folklore claim of *L. glutinosa* leaves as an antipyretic agent.

## Conclusions

In light of the results, it can be revealed that the hydroalcoholic extracts of *L. glutinosa* leaves have remarkable thrombolytic, analgesic, anti-inflammatory and antipyretic activities. Therefore, it may presage further studies to better understand the mechanism of such actions scientifically.

## Methods

### Plant material collection and identification

For the investigation, the leaves of *L. glutinosa* were collected by the authors from Potia, Chittagong, Bangladesh in July 2012. The plant was identified and authenticated by an expert botanist of Bangladesh National Herbarium (DACB), Mirpur, Dhaka (Accession No. 38277) and a voucher specimen was submitted at the herbarium for future reference.

### Extract preparation

Weighed (630 g of the dried and powdered) sample was soaked in 2200 ml of 99% methanol in clean, sterilized, and flat-bottomed glass container. Afterwards, it was sealed and maintained for 15 days accompanying occasional stirring and agitation. The complete mixture was then subjected to coarse filtration on a piece of clean, white sterilized cotton material and Whatman® filter paper. The extract was obtained by evaporation using rotary evaporator (Bibby RE-200, Sterilin Ltd., UK) at 4 rpm and 65°C temperature. It rendered a gummy concentrate of greenish color. The gummy concentrate was designated as crude extract or methanolic extract. Then the crude methanolic extract was dried by freeze drier and preserved at +4°C (yield 0.79%).The concentrated methanolic extract was partitioned by modified Kupchan method [32]and the resultant aqueous soluble partitionates i.e., n-hexane (yield approx. 19.02%), ethyl acetate (yield approx. 26.54%), and chloroform (yield approx. 6.59%) soluble fractions were used for the experimental processes.

### Chemicals

All the chemicals used in this study were of analytical grade, and purchased from Sigma Chemical Co. (St. Louis, MO, USA), and Merck (Darmstadt, Germany). To the commercially available lyophilized *S-Kinase*™ (Streptokinase) vial (Batch no: VEH 09, Popular Pharmaceutical Ltd., Bangladesh) of 1,500,000 I.U., 5 ml 0.9% sodium chloride (NaCl) was added and mixed properly. This solution was used as a stock from which 100 μl (30,000 I.U) was used for *in vitro* thrombolysis assay.

### Test animals

For the screening of *in vivo* antipyretic, analgesic, and anti-inflammatory potential of hydroalcoholic extracts of *L. glutinosa* leaves, young *Swiss-albino* mice (aged 20–25 days) of either sex, average weight 20–25 g were used. They were collected from the Animal Resources Branch of ICDDR, B (International Centre for Diarrheal Disease and Research, Bangladesh). After collection, they were kept in favorable condition for one week for adaptation and fed rodent food and water *ad libitum* formulated by ICDDR,B. Throughout the experiments, all animals received human care according to the criteria outlined in the ‘Guide for the Care and Use of Laboratory Animals’, 8th edition, prepared by the National Academy of Sciences and published by the National Institute of Health (US).

### Thrombolytic assay

*In vitro* clot lysis activity of the leaves was carried out according to the method of Prasad *et al.* [33] with minor modifications. With ethical considerations, and aseptic precaution, 7 ml of venous blood was drawn from healthy volunteers (*n* = 5) having no history of smoking, taking lipid lowering drugs, oral contraceptive or anticoagulant therapy and transferred to different pre weighed sterile micro-centrifuge tube (1 ml/tube). The micro-centrifuged tubes were subjected to incubation at 37°C for 45 min. After the formation of clot, serum was completely removed from the tubes (carried out without disturbing the clot formed) and each tube having clot was again weighed to determine the weight of the clot (clot weight = weight of clot containing tube – weight of tube alone).

To each micro-centrifuge tube containing pre-weighed clot, 100 μl solution of different extracts (n-hexane, ethyl acetate, chloroform and methanolic extract), concentration 1 mg/mL, were added accordingly. As a positive control, 100 μl of streptokinase and as a negative non thrombolytic control, 100 μl of sterilized distilled water were separately added to the control tubes numbered. Then all the tubes were incubated again at 37°C for 90 min and observed for clot lysis. After incubation, the obtained fluid was removed from the tubes and they were again weighed to observe the difference in weight after clot disruption. At last, difference obtained in weight was calculated and the result was expressed as percentage of clot lysis following the underneath equation.


### Analgesic activity test

In the current investigation two different methods were employed for testing the possible peripheral and central analgesic effects of *L. glutinosa* leaves; namely acetic acid induced writhing test and hot plate test in mice respectively.

### Hot plate test

The hot plate test was performed following the method of Asongalem *et al.* [34]. Pain reflex in response to the thermal stimulus was measured using a Le7406 hot plate (Panlab S2, Cornella, Barcelona, Spain). Before 30 min of the test, mice were placed in ten different groups comprising six (6) mice in each group and were intragastrically treated in the following manner: group I was treated with negative control (isotonic saline solution, 0.9%), group II with positive control (ketorolac) as reference drug (10 mg/kg body weight), and groups III–X with the plant extracts at dose of 250 and 500 mg/kg body weight. Mice were placed on a 55 ± 1°C hot plate in order to obtain their response to electrical heat-induced nociceptive pain stimulus judged by the presence of behaviors such as licking of the fore and hind paws or jumping. The pain response was measured at 30 min and at every hour thereafter for 4 h. The cut-off time used to prevent skin damage was 25 sec.

### Acetic acid induced writhing test

The analgesic activity of the crude methanolic extract was studied using acetic acid induced writhing model in mice [10, 35]. The animals were divided into four groups including control (Group I), positive control (Group II) and two test groups (Group III-IV). The animals of group III and IV were administered test substance at the dose of 250 and 500 mg/kg body weight respectively. Positive control group received ketorolac (standard drug) at the dose of 10 mg/kg body weight and vehicle control group was treated with 1% Tween 80 in distilled water at the dose of 10 ml/kg body weight. Test samples, standard drugs and control vehicle were administered orally 30 min before intraperitoneal administration of 0.7% acetic acid. After 15 min of time interval, the writhing (constriction of abdomen, turning of trunk and extension of hind legs) was observed on mice for 5 min.

### Anti-inflammatory activity test

Carrageenan induced mice hind paw edema was used as the animal model of acute inflammation according to the method of Lanhers *et al*. [36]. In this experiment, the mice were divided into four groups of five animals each. Group I (control) received 2% Tween 80 in normal saline (2 ml/kg). Group II (Positive control) received 10 mg/kg body wt. of ketorolac orally. Group III and IV received 250 and 500 mg/kg body wt. of the extract orally respectively. Acute inflammation was induced in all the four groups by sub plantar injection of 0.05 ml of its suspension of Carrageenan with 2% Tween 80 in normal saline in the right Paw of the mice 30 minutes after the oral administration of the tested materials. The paw volume was measured with a micrometer screw gauze at 1, 2, 3 and 4 h after the administration of the drug and the extract. The percentage inhibition of inflammatory effect of the extract was calculated using the following expression:


Where Vc is the average degree of inflammation by the control group and Vt is the average degree of inflammation by the test group.

### Antipyretic activity test

The antipyretic activity was evaluated by Brewer’s yeast induced pyrexia in experimental animal [37]. Hyperpyrexia was induced by subcutaneous administration of 10 ml/kg body weight 20% aqueous suspension of brewer’s yeast. The selected animals were fasted overnight with water *ad libitum* before the experiments. Initial rectal temperature of animals was recorded using an Ellab thermometer (33.19 ± 0.40°C). After 18 h of subcutaneous administration, the animals that showed an increase of 0.3–0.5°C in rectal temperature were selected for the antipyretic activity. Crude methanolic extract of plant was given orally (500 mg/kg). Paracetamol (150 mg/kg orally) was used as reference drug, whereas, control group received distilled water (10 ml/kg) only. The rectal temperature was recorded at 1 h intervals for 3 h after treatment [38].

### Statistical analysis

One way ANOVA with Dunnett’s post Hoc test for this experiment was carried out with SPSS 16.0 for Windows® software and the results obtained were compared with the control group. P values <0.001 were considered to be statistically significant.
